# Visible light-induced thymine dimerisation based on large localised field gradient by non-uniform optical near-field

**DOI:** 10.1038/s41598-019-54661-6

**Published:** 2019-12-05

**Authors:** Naoya Tate, Takashi Yatsui

**Affiliations:** 1 Kyushu University, 744 Motooka, Nishi-ku, Fukuoka 819-0395 Japan; 2 The University of Tokyo, 7-3-1 Hongo, Bunkyo-ku, Tokyo 113-8656 Japan

**Keywords:** DNA nanotechnology, Optical techniques

## Abstract

The localised excitations of several molecular reactions utilising optical irradiation have been studied in the field of molecular physics. In particular, deoxyribonucleic acid (DNA) strands organise the genetic information of all living matter. Therefore, artificial methods for freely controlling reactions using only light irradiation are highly desirable for reactions of these strands; this in regard with artificial protein synthesis, regional genetic curing, and stochastic analysis of several genetic expressions. Generally, DNA strands have strong absorption features in the deep ultra-violet (DUV) region, which are related to the degradation and reconstruction of the strand bonding structures. However, irradiation by DUV light unavoidably induces unintended molecular reactions which can damage and break the DNA strands. In this paper, we report a photo-induced molecular reaction initiated by the irradiation of DNA strands with visible light. We utilised photo-dissociation from the vibrational levels induced by non-uniform optical near-fields surrounding nanometric Au particles to which DNA strands were attached. The results were experimentally observed by a reduction in the DUV absorbance of the DNA strands during irradiation. There was a much higher yield of molecular reactions than expected due to the absorbance of visible light, and no defects were caused in the DNA strands.

## Introduction

Deoxyribonucleic acid (DNA) strands consist of combinations of four nucleobases, adenine (A), guanine (G), cytosine (C), and thymine (T), the sequence of which determines the genetic information of living matter. The development of artificial techniques for controlling the molecular reactions of DNA strands is a fundamental issue for experimental studies on artificial protein synthesis, regional genetic curing, and stochastic analysis of several genetic expressions^1,2^. The photo-excitation method for such molecular reactions is one of the most useful techniques due to its technical ease and variable physical parameters^3^. Unlike higher linear energy transfer radiation such as *x*-rays and *γ*-rays, DNA strands are characterized by only a few spectral absorbance features in the ultraviolet (UV), visible, and infrared (IR) regions. In contrast, DNA absorbs a large amount of deep UV (DUV) photons at optical energies greater than 4.0 eV; these energies correspond to their base sequence and related binding structures. Thus, irradiating DNA strands with light induces the degradation and reconstruction of their binding structures and corresponding changes to their biochemical properties. Furthermore, the combination of irradiation with photo-responsive organic molecules, such as stilbenes^4^, diarylethenes^5^, spiropyrans^6^, and azobenzenes^7^, has been experimentally verified to allow for the variable control of DNA molecular reactions.

Although the above-described photo-excitation methods have valuable advantages, excessive DUV irradiation will induce some unintended reactions which can damage and break DNA strands due to electronic dissociation^8^. In this paper, we propose a photo-excitation method based on the characteristics of non-uniform optical near-fields (ONFs), which are induced through irradiation by visible light with a much lower optical energy than DUV light^9^. Because contributions from the excited vibrational levels originating from the non-uniform ONFs can compensate for the lack of activation energy for the degradation of DNA strands, we expected the non-uniform ONFs to allow for photo-excitation at a much lower optical energy, thereby avoiding the initiation of any unintended molecular reactions. As an experimental verification of our proposal, we stochastically observed the specific decreases in the absorbance of visible light of DNA strands. First, we describe the basics of the localised large-field gradient induced by non-uniform ONFs and the photo-dissociation via the vibrational levels of DNA strands due to ONF excitation. We then describe the experimental demonstrations of the proposed method, which utilised visible light irradiation of poly-thymine (poly-T) strands attached to Au nanoparticles to cause ONF-induced thymine dimerisation. Finally, we quantitatively evaluate the applicability of our proposed approach.

## Results

### ONF-induced thymine dimerisation

Although the photo-excitation of molecular reactions by DUV irradiation can induce molecule reactions at higher yields, excessive irradiation unavoidably causes unintended molecular reactions. To resolve this concern, we utilised the characteristic behaviour of non-uniform ONFs^9^. An ONF is defined as a localised electric field generated around a nano-scaled material^10^, where an induced dipole is generated by light irradiation. One of the qualitative differences between ONFs and conventional propagating light is that ONFs are characterized by a large field gradient in a nanometric space. Recently, the effects of non-uniformity on molecular photo-dissociation processes have been discussed^9^, and a theoretical investigation into the dissociation process of ${{\rm{H}}}_{2}^{+}$ induced by a non-uniform ONF^11^ was demonstrated. ${{\rm{H}}}_{2}^{+}$ is often used in experimental and theoretical studies on photo-dissociation^12–16^. It has been demonstrated that a non-uniform ONF can dissociate ${{\rm{H}}}_{2}^{+}$ through a novel two-step transition path mediated by vibrationally excited levels in the electronic ground state. This dissociation path arises from the non-uniformity of the ONF causing the excitation of higher-order vibrational states which cannot be accessed by uniform excitation with propagating light under the dipole approximation. We have reported several experiments on multi-step transitions induced by non-uniform ONFs including CO_2_ reduction^17^, water splitting^18^, and near-field etching^19^. Therefore, we hypothesized that a similar multi-step transition could be employed in thymine dimerisation.

**Figure 1:**
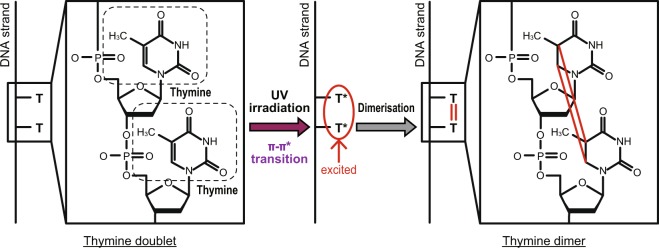
Simplified schematic of the photo-dynamics of thymine dimerisation.

Based on the above discussions, we focused on thymine dimerisation by non-uniform ONF. Thymine dimerisation^20^ is a major photo-chemically induced lesion. Two neighbouring T bases in a DNA strand, which is referred to as a TT doublet, are preferentially dimerised by DUV irradiation (Fig. 1). The formation of thymine dimers causes messenger RNA transcription errors which generally lead to a complex web of biological responses including apoptosis, immune suppression, and carcinogenesis^21–23^.

**Figure 2:**
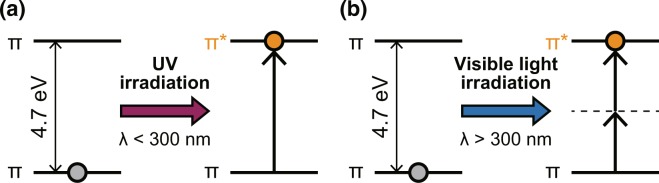
(**a**) Schematic of single-step excitation of π-π* transition by far-field light propagation with a UV photon and (**b**) multi-step excitation by a non-uniform ONF with a visible photon.

The dimerisation process is fundamentally based on electronic excitation and the corresponding orientation of the reacting double bonds in each molecule^20^. When the excitation energy of the π-π* transition is greater than 4.7 eV, irradiation with the corresponding optical energy induces the excitation of T, which is referred to as T* in Fig. 1. Subsequently, some of the excited-state population decays to form the TT dimer products. Therefore, as shown in Fig. 2(a), far-field light must be used for conventional optical dimerisation. Here, we experimentally demonstrate a novel optical dimerisation utilising vibrational excitation induced by a non-uniform ONF. As discussed in the related article by Yatsui *et al*.^9^, when the ONF is affected as shown in Fig. 2(b), the activation of vibrational excitation levels is expected, followed by multi-step excitation. In this case, light at wavelengths shorter than 300 nm can be utilised to inhibit damage to the RNA strands to the greatest extent.

Generally, photo-induced molecular reactions of DNA molecules are classified as either direct or indirect action^24^. In direct action, the optical energy of the irradiated light directly affects the dissociation and excitation of the target and induces corresponding molecular reactions. On the other hand, in indirect action, the incident optical energy first induces the generation of radicals of molecules surrounding the target, which then interact with the target. Because indirect action requires much higher optical energies, and this paper mainly discusses visible light irradiation, we herein assume the occurrence of only direct action on the DNA molecules for simpler discussions.

### Analysis of absorbance characteristics

The reaction yields by photo-excitation were primarily verified by the changes in the absorbance of the DNA strands. Generally, the absorbance features of DNA strands are mainly in the DUV region, which corresponds to energies higher than 3.2 eV. The combination of the four nucleobases which construct DNA and their related bonding energies determine these absorption energies. Thus, the degradation and reconstruction of these bonding structures during particular molecule reactions will unavoidably affect the absorption features.

As shown in Fig. 1, the cleavage of double bonds in each T base and subsequent covalent bonding between the two occurs by dimerisation. In the dimerisation process, the absorbance features gradually change due to the bonding structure of each T base. In paricular, the absorption peak at a wavelength of approximately 267 nm, which corresponds to the double-bonded structure within the T pyrimidine ring, decreases significantly upon dimerisation^17^. Furthermore, (6–4) photoproducts are also obtained as dimerisation by-products^25^ due to the bonding of a TT doublet by a single bond. This absorption peak occurs at approximately 325 nm, differing from that of the dimer product. Finally, by further irradiation, all strands, including the thymine dimers and (6–4) photoproducts, are degraded, and the corresponding absorption peaks decrease. In our experiments, the absorption spectra of each solution were observed using a spectrophotometer (U-3000, Hitachi High-Technologies, Japan).

### Irradiation of DUV light

**Figure 3:**
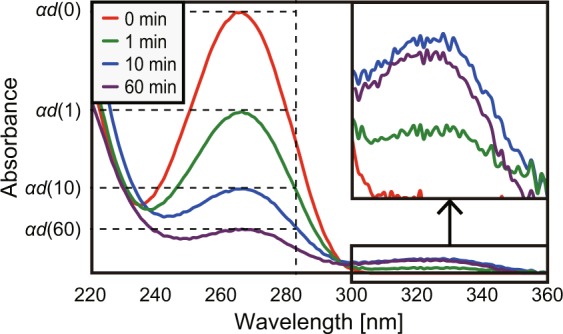
Changes in absorption peaks due to produced thymine dimers and (6–4) photoproducts during DUV irradiation.

Prior to the demonstration of dimerisation by a non-uniform ONF, we first verified the decrease in the absorption peak of poly-T molecules caused by DUV irradiation. Laser diodes (LDs) at a wavelength of 266 nm were used as a DUV light source, and the power of the LDs was set at 3 mW. Figure 3 shows the changes in the absorption spectra during irradiation and the decrease in the peak intensity at 267 nm.

**Figure 4:**
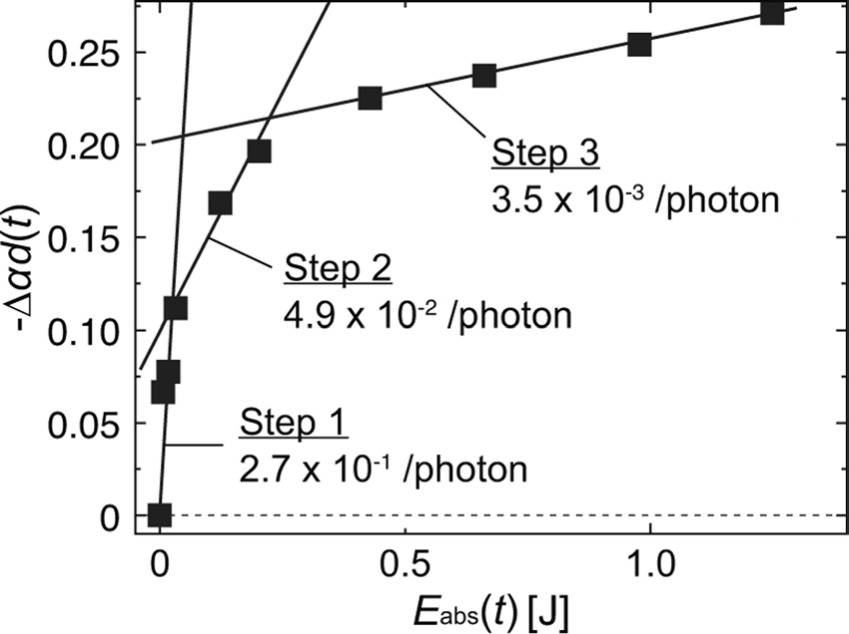
Relationship between intensity decrease −Δ*αd*(*t*) and absorbed optical energy *E*_abs_(*t*) depending on irradiation at 266 nm.

The absorption peak at 267 nm gradually decreased during irradiation, which allowed for indirect monitoring of the dimerisation process. Moreover, the absorption peak behaviour at approximately 325 nm was also observed, where the peak intensity increased until 10 min of irradiation. The origin of this peak has been demonstrated as the formation of (6–4) photoproducts^25^. Another important finding is that more than 60 min of DUV irradiation induced the degradation of the poly-T strands, which resulted in a decrease in the absorption peak intensities. Here, we quantitatively define this decrease in intensity as −Δ*αd*(*t*), which was calculated as the difference in intensities between the peaks at 0 and *t* min of light irradiation, *αd*(0) and *αd*(*t*), respectively. The corresponding optical energy, *E*_abs_(*t*), absorbed during irradiation was defined as *E*_abs_(*t*) = (1–10^−Δ*αd*(*t*)^)*It*, where *I* represents the power of the irradiated light. The resulting absorption peak decrements, −Δ*αd*(*t*), shown in Fig. 3 are plotted as a function of *E*_abs_(*t*) (Fig. 4).

As shown in Fig. 4, the absorption intensity decrease can be fitted by three lines with slopes of 2.7 × 10^−1^, 4.9 × 10^−2^, and 3.5 × 10^−3^/photon, respectively. Here, each slope corresponds to the quantum efficiency of the stepwise molecular reactions. The *E*_abs_(*t*) values of the third line correspond to 60~240 min of laser irradiation, where the poly-T strands were degraded as indicated in Fig. 3. The behaviours of the poly-T strands during the subsequent reactions can be assumed as follows:

Step 1. Random dimerisation occurs between arbitrary TT doublets in the poly-T strands.

Step 2. Dimerisation is suppressed due to the decrease in remaining TT doublets, and (6–4) photoproducts are additionally formed.

Step 3. Dimerisation is saturated, and the poly-T strands are damaged and broken by the absorbance of excess optical energy.

### Irradiation of visible light

**Figure 5:**
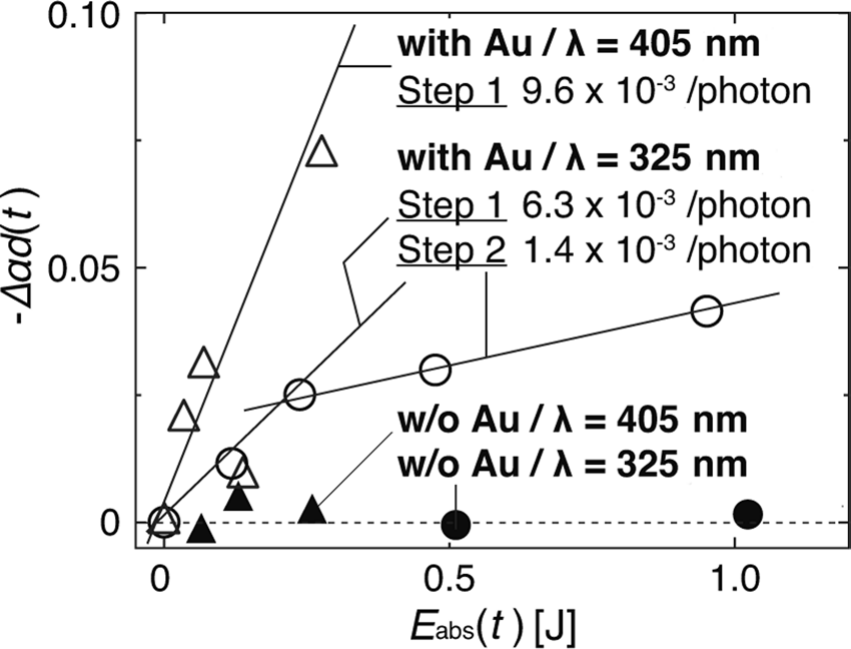
Relationship between −Δ*αd*(*t*) and *E*_abs_(*t*) depending on irradiation at 325 and 425 nm.

To experimentally demonstrate dimerisation with a non-uniform ONF, LDs at wavelengths of 325 and 405 nm were used to irradiate two sample solutions, one containing poly-T bonded to Au nanoparticles and the other containing only poly-T strands, respectively, to verify the involvement of a non-uniform ONF in the vicinity of the Au nanoparticles. The changes in absorption were monitored in a manner similar to that used for DUV irradiation, where the optical powers were set to 25 and 100 mW, respectively. Figure 5 shows the decreases in intensity induced by irradiation with 325-nm and 405-nm light. To eliminate the absorbance of the Au nanoparticles from the results, differential absorption spectroscopy was performed by substituting the absorbance from the measured results.

**Figure 6:**
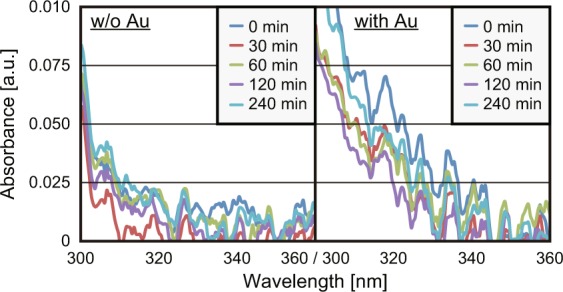
Absorbance of poly-T strands from 300 to 360 nm during irradiation at 325 nm with (right) and without (left) bonded Au nanoparticles.

As shown in Fig. 5, only the absorbance peaks of the poly-T strands bonded to Au nanoparticles (white triangles and circles) clearly decreased in intensity. In addition, it was found that the results could be fitted by only two lines rather than three as shown in Fig. 4 with similar amounts of *E*_abs_(*t*) to the DUV irradiation experiment. These results indicate that there was no direct effect of irradiation or any molecular reactions induced by visible light. On the other hand, with the bonded Au nanoparticles and associated non-uniform ONF assumed to surround the particles due to irradiation at 325 and 405 nm, the dimerisation process occurred as it did under DUV irradiation. However, because the process consists of only two steps without Step 3 in Fig. 4, it is assumed to not involve any damaging or breaking of poly-T strands, as we expected. As evidence of our assumption, Fig. 6 shows the absorbance of the poly-T strands during irradiation by 325-nm light.

## Discussion

As shown in Fig. 6, there is no clear peak at approximately 325 nm regardless of the presence of Au nanoparticles bonded to the DNA strands. This means that 240 min of visible light irradiation cannot induce the formation of (6–4) photoproducts or the subsequent damaging and breaking of strands.

The difference in the quantum efficiencies between irradiation at 325 and 405 nm is assumed to be due to the absorption edge of the Au nanoparticles. Namely, 325 nm is much closer to the Au absorption edge of approximately 300 nm, and thus more optical energy interacts with the Au particles to generate a larger ONF. For this reason, irradiation of 325-nm light reached Step 2 and converged more rapidly than udner 405-nm irradiation.

We demonstrated the photo-excitation of molecular reactions induced by irradiation with a lower optical energy than the absorption band edge by introducing a non-uniform ONF. In our experiment, a number of poly-T strands were bonded to Au nanoparticles as an ONF source to clarify the dimerisation process. The results indicate an evident amount of dimerisation according to changes in the absorption spectrum, which occurred only for the strands bonded to Au nanoparticles. This suggests that the multi-step excitation of π-electrons and subsequent dimerisation due to the ONF occurred successfully. Moreover, the results indicate that no damaging or breaking of the poly-T strands occurred despite the application of a similar amount of optical power to that used for DUV irradiation. This is a significant and useful potential method for the artificial and localised control of genetic expressions based on the biochemical reactions of DNA strands without causing any defects to the target strands.

## Methods

### Preparation of poly-T strands

In our experiments, commercially available poly-T strands consisting of 60 T bases were prepared to maximise the decrease in absorbance intensity due to dimerisation. The length of the poly-T strands was assumed to be 20 nm, and each strand was thiol-terminated. Each experiment utilised 200 μL of sample solution with a 0.32 μM final density of poly-T strands. These diluted solutions were used to avoid absorbance saturation.

### Bonding of poly-T strands and Au nanoparticles

**Figure 7:**
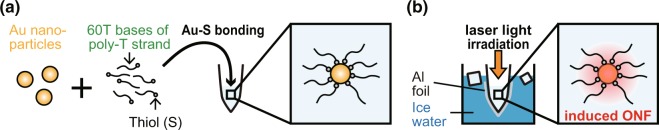
Schematic diagram of experimental process: (**a**) bonding of poly-T strands to Au nanoparticles as the ONF source; (**b**) excitation of ONF by irradiation of light.

For the experiments with a non-uniform ONF, the ONF source must be prepared in the vicinity of the poly-T strands. In this experiment, as shown in Fig. 7(a), a number of poly-T strands were attached to Au nanoparticles by Au-thiol bonding, which is known to be highly stable bonding which cannot be dissociated by irradiation. In this case, the Au nanoparticles acted as the ONF source. Here, the average diameter of the applied Au nanoparticles was 20 nm. In our experiment, more than 500 poly-T strands were assumed to be bonded to each Au nanoparticle. To effectively begin the bonding process, a 5.0 nM Au nanoparticle solution and 2.5 μM poly-T solution were mixed. Then, after leaving the mixture at room temperature for one day, it was diluted eightfold by distilled water and 200 μL of the prepared sample solution. The mixing status was experimentally identified by electrophoresis, which migrates un-bonded poly-T strands.

### Laser light irradiation

The sample solution in a micro-tube was directly irradiated by laser light. Because the spatial distribution of the ONF is approximately defined to be similar in size to that of the nano-structure of the source^9^, the ONF was expected to be distributed over the bonded 60 T bases of the poly-T strands as shown in Fig. 7(b). In order to avoid the effects of heating by laser irradiation, the sample micro-tube was covered by Al foil and fully immersed in ice water during irradiation.
